# Synthesis of Electrolytic Manganese Slag–Solid Waste-Based Geopolymers: Compressive Strength and Mn Immobilization

**DOI:** 10.3390/ma17061431

**Published:** 2024-03-21

**Authors:** Bao Mi, Hui Zhao, Meng Lu, Yi Zhou, Yongjie Xue

**Affiliations:** State Key Laboratory of Silicate Materials for Architectures, Wuhan University of Technology, Wuhan 430070, China

**Keywords:** electrolytic manganese residue, coal gangue, geopolymer, compressive strength, Mn, immobilization

## Abstract

The massive stockpiling of electrolytic manganese residue (EMR) has caused serious environmental pollution. In this study, EMR, coal gangue (CG), and fly ash (FA) were used as raw materials to obtain the optimal mix ratio based on Design-Expert mixture design. The effects of activator modulus, liquid–solid (L/S) ratio, and curing temperature on the mechanical properties of geopolymers were investigated. The results showed that the compressive strength of the prepared geopolymer was 12.0 MPa, and the 28d leaching of Mn was 0.123 mg/L under the conditions of EMR:CG:FA = 0.43:0.34:0.23, L/S = 0.9, a curing temperature of 60 °C, and a curing time of 24 h. This indicates that the geopolymer is an environmentally friendly material with high compressive strength. The mineral composition of the geopolymer is mainly hydrated calcium silicate and geopolymer gel. In addition, a more stable new mineral phase, MnSiO_3_, was generated. The Fourier transform infrared (FTIR) spectrogram showed that the peak at 1100 m^−1^ was shifted to 1112 cm^−1^, which indicated that a geopolymerization reaction had occurred. Through scanning electron microscopy (SEM) and energy dispersive spectrum (EDS) analysis, it was identified that the geopolymerization produced a large amount of amorphous gelatinous substances with a relatively dense structure, the major elements being oxygen, silicon, aluminum, calcium, and sodium.

## 1. Introduction

Electrolysis Manganese Residue (EMR) is the waste residue generated during solid–liquid separation in the filtering process of electrolytic production of manganese metal [1]. At present, there are about 160 million tons of untreated electrolytic manganese slag in China, and the rate of increase is 10 million tons per year [2], which not only occupies a large amount of land resources, but also causes serious pollution to the surrounding soil and water due to the presence of a large number of heavy metals in manganese slag, which can easily leach out and enter into the soil, surface water, or groundwater under the action of rainfall [3]. With the introduction and implementation of a series of policies, such as the Action Program for In-Depth Protection and Restoration of the Yangtze River and the Opinions of the CPC Central Committee–State Council on In-Depth Prevention and Control of Pollution, the pollution problem of EMR is a problem that restricts the sustainable development of the manganese electrolysis industry, and it is also the key to realizing ecological governance within the country. Therefore, there is a need to render EMR non-hazardous and resourceful. The harmless treatment of electrolytic manganese slag mainly includes water washing [4], acid leaching [5], electrokinetic restoration [6], solidification/stabilization (solidification/stabilization, S/S) technology [7], and other routes. The first three methods have high processing costs and complex technical routes, with scholars now favoring efficient, energy-saving, easy-to-operate S/S technology; compared with other S/S technologies, geopolymers have the advantages of fast setting and early strengthening, good durability, inexpensive costs, and low carbon emissions, which have attracted the attention of researchers [8,9,10].

Geopolymer is a kind of inorganic, highly polymerized, cementitious material prepared using an alkali activator with aluminosilicate raw materials (ASMs) as the raw material, and the physical form is a three-dimensional network gel with amorphous and quasi-crystalline characteristics [11]. Geopolymer has developed significantly, and the choice of raw materials and activators has broadened considerably, with a wide range of sources of ASMs for the preparation of geopolymers that extends from kaolinite [12] to fly ash (FA) [13], steel slag [14], sludge [15], coal gangue (CG) [16], and other solid wastes [17]. EMR has high silica–aluminum content and is a potential ASM for the preparation of geopolymer. The preparation of geopolymer from EMR can solve the environmental problems caused by EMR and realize the harmless treatment of EMR, but only using EMR as a raw material cannot provide enough ASM for the geopolymerization reaction. Numerous studies have shown that CG and FA are good silica–aluminum feedstocks for the preparation of geopolymer materials and can replace a portion of EMR to prepare geopolymer with excellent properties [18]. Wang et al. [19] produced a geopolymer with a 28d strength of 22 MPa through alkaline activation of uncalcined gangue and fly ash at a ratio of 3:7. Beata Figiela et al. [20] used mining wastes such as gangue to prepare geopolymers, exploring the possibility that these solid wastes could be used as materials in the wider construction industry. Liet al. [21] calcined electrolytic manganese slag mixed with sodium hydroxide at a mass ratio of 1:0.2 at 200 °C, then calcined with fly ash at a mass ratio of 3:2. After mixing with fly ash at the ratio of 3:2, a geopolymer of 16 MPa was produced at 15 MPa, which demonstrated good environmental stability and leaching toxicity in accordance with the national standard. Meanwhile, the related experiments showed that the geopolymer had better performance in heavy metal curing [22,23,24]. In recent years, even though there have been many attempts to use EMR, the consumption of EMR is still relatively low [25]. There are fewer studies on the use of higher dosages of EMR or the preparation of cementitious materials with high dosages of EMR without the aid of pressure molding. The aim of this study was to increase the percentage of EMR in the raw material without using pressure molding. To achieve more efficient treatment of EMR and alleviate the environmental problems caused by EMR stockpiling, the use of pour molding can lower energy consumption and reduce carbon emissions. It provides a new idea for the harmless and resourceful utilization of EMR.

In this study, a large amount of manganese waste residue produced in the manganese electrolysis industry, waste gangue produced in coal mining, and fly ash were utilized as raw materials. Geopolymers were prepared by cast molding, i.e., without using applied pressure molding. The mixing ratio and optimization were designed using Design-Expert software (V8.0.6.1), which can avoid the disadvantage of orthogonal experiments that cannot provide visual images. The best mixing ratio was then derived under the condition of disposing of as much EMR as possible. The effects of the modulus of alkali activation solution, the dosage of alkali activation solution, and the curing conditions on the geopolymers were then investigated. In addition, the performance of electrolytic manganese slag–solid waste-based geopolymer (SW-GP) was evaluated by analyzing the mineral phase, morphology, and leaching concentration of the geopolymer.

## 2. Experimental Section

### 2.1. Materials

EMR with a moisture content of 14% was taken from a manganese electrolysis enterprise in Guizhou, China, the CG was taken from Yangquan, Shanxi, and the FA used in this study belonged to low-calcium fly ash and was purchased from Wuhan, China, from a materials company. The physical and chemical properties of EMR, CG, and FA are listed in Table 1. The silica–alumina content of CG and FA, containing more than 80% of the sum, is very suitable as a raw material for the preparation of geopolymers, and the silica–alumina content of EMR is about 40%, which has the potential to be used as a raw material for the preparation of geopolymers. Sodium hydroxide (NaOH) particles were purchased from Sinopharm Reagent (Shanghai, China), and sodium silicate (Na_2_SiO_3_) powder with a modulus of 3.14 was purchased from Aladdin Co. (Shanghai, China) All reagents were analytically pure reagents.

### 2.2. Preparation of EMR–Solid Waste-Based Mass Polymers

**Figure 1:**
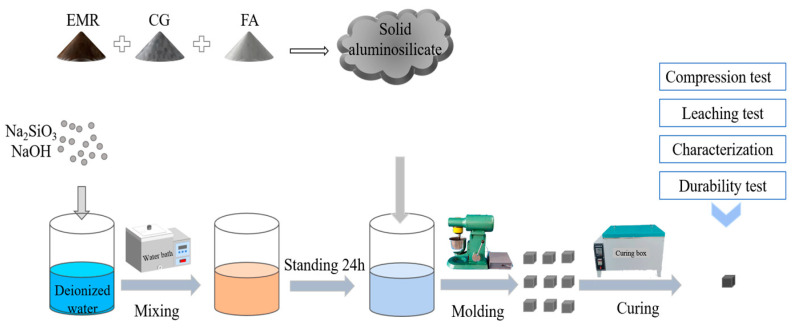
Schematic demonstrating preparation of EMR–solid waste-based geopolymer.

A schematic diagram of the preparation of geopolymer is shown in Figure 1. (1) EMR, CG, and FA were all dried in an oven at 105 °C for 4 h and ground in a ball mill for 60 min before being cooled to room temperature; the materials were sieved using a 100 mesh sieve before being set aside. (2) The solid Na_2_SiO_3_ powder with a modulus of 3.14 was dissolved in distilled water, and sodium hydroxide was then added to obtain a sodium silicate solution with a modulus of 1.5 (60% H_2_O, 24% SiO_2_, 16% Na_2_O) which was then allowed to cool to room temperature. (3) The results show that the geopolymer slurry has better fluidity when the liquid–solid (L/S) ratio is 0.9 and is suitable for casting. (4) The mixing experimental design was designed according to Table 2, and the EMR, CG, and FA were then weighed according to the proportion in Table 3 before being poured into a planetary stirring pot and stirred for 2 min, followed by the addition of alkali exciters according to the L/S mass ratio of 0.9, stirring at a low speed for 2 min, and stirring at a high speed for 3 min to obtain a black slurry which was then poured into a 20 mm × 20 mm × 20 mm mold. (5) After the gas in the slurry was expelled by the shaker, it was placed in an incubator with a constant temperature of 60 °C and humidity of 96% to cure for 24 h and delaminate to obtain the final geopolymers. The resulting specimens were then cured in the incubator for 1d to obtain the final geopolymer. Finally, the samples were placed in sealed bags and stored at room temperature, ready for compressive strength testing and other characterization at the appropriate age.

The mix ratios of the mixes were designed using Design-Expert software. The contents of EMR, CG, and FA were set as independent variables and compressive strength was selected as a dependent variable. D-optimal mixes were then chosen in the design of the response surface test, and the interactions of EMR, CG, and FA were then analyzed to establish a response surface of the proportion of raw materials and the mechanical strength of the geopolymer. The optimal response surface model was derived by analyzing the fit of the surface’s model, and the optimization conditions were finally set to derive the optimal fit ratio. The experimental factorial design values are shown in Table 2. It is found that when the amount of EMR is greater than 55%, the geopolymer has low strength and cannot be molded smoothly, and when the amount of EMR is less than 15%, the mechanical strength of the geopolymer is mainly provided by CG and FA, which is of less value for the present study.

### 2.3. Test Methods

#### 2.3.1. Compressive Strength

The compressive strength test was carried out using a TYE-3000 compression testing machine (Wuxi Jianyi Instrument & Machinery Co., Ltd., Wuxi, China). The samples were 20 × 20 × 20 cm cubes, and the loading rate was 0.3 MPa/s. A total of 3 specimens were tested in each group and the average value was taken [26]. Strength was compared with that of standard no-burn bricks [27]. Compressive strength was calculated according to Equation (1).

(1) $$R_{c}=\frac{F_{c}}{A}$$

where *R*_c_ (MPa) is the compressive strength of the geopolymer and *F*_c_ (N) is the maximum pressure on the geopolymer. *A* (mm^2^) is the pressurized area of the geopolymer, which is 4 × 10^2^ mm^2^ in this study.

#### 2.3.2. Leaching Toxicity

The toxicity leaching experiments of geopolymers were conducted according to the solid waste–leaching toxicity–horizontal oscillation method (HJ/T 557-2010) [28]. A Labs Prodigy7 plasma emission spectrometer (Prodigy7, Leeman Labs, Hudson, NH, USA) from Leeman Labs was used to determine the concentration of heavy metals in the leachate; a Lambda 750S ultraviolet-visible near-infrared spectrophotometer (Lambda 750S, PerkinElmer, Waltham, MA, USA) from PerkinElmer was used to test the leachate’s ammonia nitrogen content.

The leaching concentrations of the samples were measured and compared with the limits of heavy metals in leachate (total Mn < 2 mg/L) and ammonia nitrogen in leachate (ammonia nitrogen < 15 mg/L) according to hazardous waste leaching toxicity identification criteria (GB5085.3-2007) [29].

### 2.4. Characterization

The composition of the raw materials was analyzed using an X-ray fluorescence (XRF) spectrometer (Zetium, PANalytical B.V., Almelo, The Netherlands). The physical phases of the raw materials and geopolymers were analyzed using an X-ray diffractometer (XRD) (D/Max-RB, Rigaku, Tokyo, Japan). Surface morphology was analyzed by scanning electron microscopy (SEM) (JSM5610, JEOL, Akishima, Japan) and energy dispersive spectroscopy (EDS) at an accelerating voltage of 5 kV. A Fourier transform infrared (FTIR) spectrometer (Nexus, Thermo Nicolet, Waltham, MA, USA) was used. The EMR and geopolymer surfaces, the chemical states of O, Si, Al, and Mn, and molecular structural information were analyzed using an X-ray photoelectron (XPS) spectrometer (ESCALAB 250Xi, Thermo Fisher, Waltham, MA, USA), and all peaks were corrected using C-C (284.8 eV).

## 3. Results and Discussion

### 3.1. D-Optimal Mix Design

#### 3.1.1. Analysis of Mixing Results

**Figure 2:**
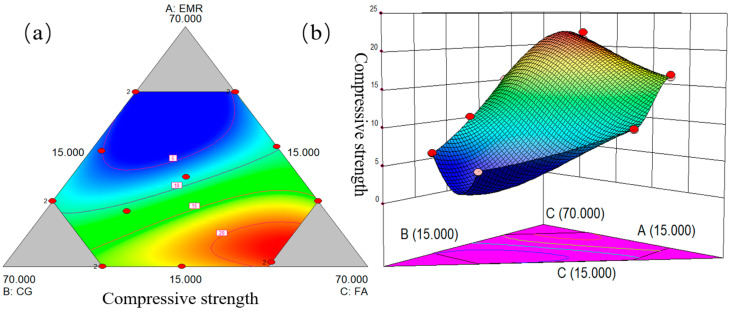
(**a**) Planar response plot of compressive strength. (**b**) Three-dimensional response plot of compressive strength.

The compressive strength of each group of samples is shown in Table 3. It is clear from Figure 2 that the contents of EMR, CG, and FA all have a significant effect on compressive strength. Figure 2 shows that the response surface curves show a slow increase with increasing FA content, indicating that compressive strength is positively correlated with the proportion of FA, i.e., the more FA is added, the greater the compressive strength of the samples. On the other hand, EMR content was negatively correlated with the relative range of strength.

The experimental data were processed using Design-Expert software to fit the different models non-linearly. The results show that the special quadratic model can better respond to the mathematical relationship between the compressive strength of geopolymers and the EMR, CG, and FA variables. The specific regression model expression formulas are shown in Table 4.

**Figure 3:**
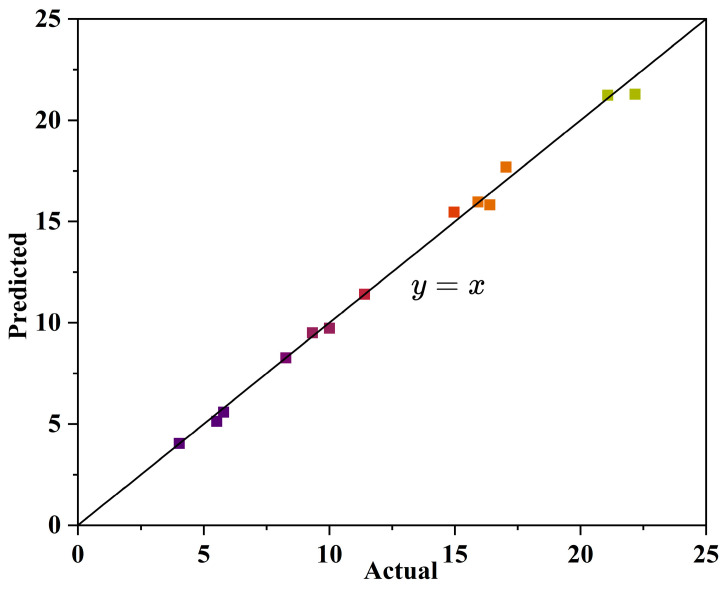
Predicted and actual values of compressive strength.

The reliability of the model was tested using R^2^, with higher R^2^ values indicating higher reliability of the model. The regression coefficient (R^2^) of the model’s compressive strength was 0.9922, indicating that the model was in good agreement with the experimental data. ANOVA was used to test the significance of the model and the results are shown in Table 5. When the F value is greater than the *p* value and the *p* value is less than 0.05, the independent variable is statistically significant at the 95% confidence level, while when the *p* value is less than 0.01, the independent variable is statistically significant at the 95% confidence level. As shown in Table 4, the model’s fitted compressive strength F value is greater than 1, indicating that the model contributes more variance than the random error. The *p* value for compressive strength is less than 0.01, indicating that the regression effect is highly significant. Figure 3 shows the results of the line fitting of the predicted and actual values of the model. With the straight line y = x as the 0 error line, the X values corresponding to each point are the actual values and the Y values are the predicted values. It can be found that the predicted and actual values basically conform to the line fit, indicating that the data model is real and effective.

#### 3.1.2. Optimization and Validation

Based on the use of a geopolymer system for multiple disposal of EMR, reductions in the amount of FA, compressive strength with reference to MU7.5 in the General Specification for Masonry Structures, the overall design of the test, as well as optimization of the data from the Design-Expert software, the predicted values of the mathematical simulation and optimization ratios were obtained, as shown in Table 6. In terms of prior studies, Zhan et al. [24] used 75 wt% FA and 25 wt% EMR to prepare geopolymer to cure heavy metals. Wang et al. [30] (10% doped EMR) replaced a portion of FA with 10% EMR to prepare a gelling material with a strong adsorption effect on Mn^2+^. In contrast, in this study, the optimized mix ratio using Design-Expert software, in which the doping of EMR was 43%, was used to prepare geopolymer, which could achieve the purpose of utilizing EMR in large quantities.

The geopolymers were prepared according to the proportion in Table 6 (EMR:CG:FA = 0.43:0.34:0.23), and strength tests were conducted and after 1d of curing, with compressive strength at day 1 7, and 28 of 6.9 MPa, 7.5 MPa, and 8.4 MPa, respectively. It is clear that the compressive strength of the geopolymer increases gradually with curing time, though the 1d strength reaches 82% of the 28d strength, which may be due to the fact that the higher calcium content in the EMR promotes the dissolution of the ASM and accelerates gel formation, leading to faster solidification [31].^.^

### 3.2. Effect of the Preparation Process on Geopolymers

**Figure 4:**
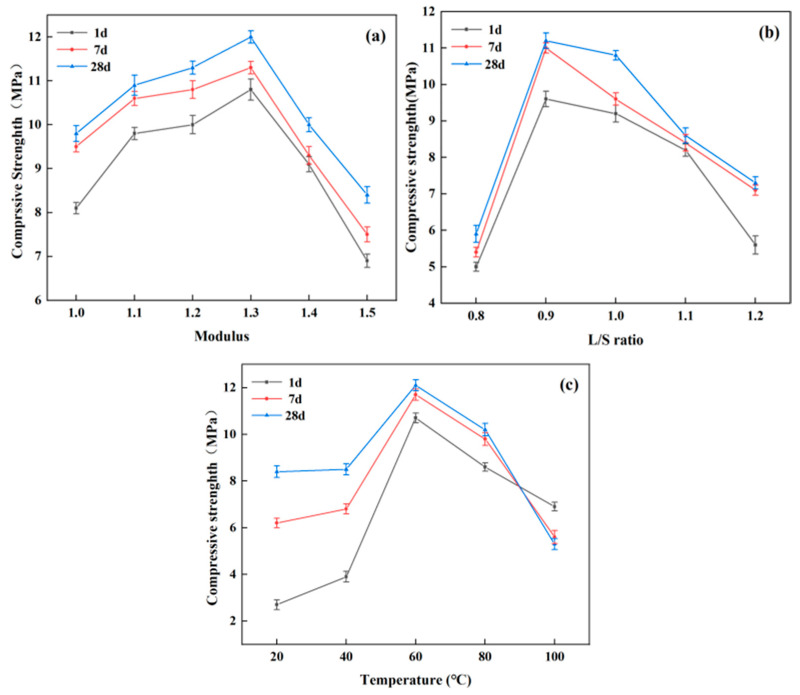
(**a**) Effect of modulus on the compressive strength of geopolymers. (**b**) Effect of L/S ratio on the compressive strength of geopolymers. (**c**) Effect of temperature on the compressive strength of geopolymers.

Geopolymer specimens were prepared using the coordination ratio of EMR:CG:FA = 0.43:0.34:0.23 to investigate the effect of the preparation process on the strength of geopolymer. Figure 4 shows the effect of activator modulus, L/S ratio, and temperature on the compressive strength of geopolymer at day 1, 7, and 28. A specific analysis of each parameter is given below.

#### 3.2.1. Activators Modulus

From Figure 4a, it can be seen that compressive strength first increases and then decreases with the increase in activator modulus and reaches the maximum at a modulus of 1.3, with strength at day 1, 7, and 28 of 10.0 MPa, 10.8 MPa, and 10.9 MPa, respectively. The increasing trend in compressive strength is mainly due to the increase in sodium content in the mixture, and Na^+^ acts as a charge-balancing ion that promotes the growth of crystals in the hydration products and plays a key role in the formation of geopolymers [32]. When the alkali activator modulus is too high, the pH of the liquid-phase environment of the geopolymerization system decreases and the polymerization degree of SiO_2_ increases, with the higher polymerization degree of SiO_2_ possibly requiring further activation in order for participation in the geopolymerization reaction to occur. A decrease in pH is not only detrimental to the dissolution of ASM, but also reduces the further activation of the high polymerization degree of SiO_2_ in the excitant, which results in a decrease in the strength of the geopolymer [33]. Therefore, it is not a case of the higher the better for the activator modulus, and it is better to choose activators with a modulus of 1.3 for the preparation of geopolymer.

#### 3.2.2. L/S Ratio

The influence of L/S ratio on the compressive strength of geopolymer is shown in Figure 4b. As the L/S ratio increases, the compressive strength of the geopolymer first increases and then decreases while reaching a maximum at an L/S ratio of 0.90. Strength at day 1, 7, and 28 was measured as 9.6 MPa, 11.0 MPa, and 11.2 MPa, respectively. On the one hand, a low L/S ratio prevents the ASM from being adequately dissolved and hinders the geopolymerization reaction, resulting in low compressive strength. On the other hand, if the L/S ratio is too high, the excess water will be discharged from the specimen and holes will be formed at the water flow, leading to a reduction in the compressive strength of the specimen [34]. Therefore, an L/S ratio of 0.9 was selected as the best ratio.

#### 3.2.3. Temperature

As shown in Figure 4c, the compressive strength of geopolymer increases and then decreases with the increase in conservation temperature and reaches the maximum at 60 °C, with strength at day 1, 7, and 28 measured as 10.7 MPa, 11.7 MPa, and 12.0 MPa, respectively. When the temperature is too low, the dissolution rate of the ASM component is slow and the hydration is not rapid enough, which prevents the geopolymer structure from being close enough and leads to a decrease in the strength of the geopolymer [35]. When the temperature is too high, the polymerized structure of the geopolymer is disrupted, leading to dehydration, excessive shrinkage, and microcracks, which reduce strength [36]. Therefore, it is very important to choose a suitable temperature for geopolymers. It can be concluded that better mechanical properties can be obtained by selecting 60 °C for the curing of geopolymers.

### 3.3. Leaching Toxicity

The leaching contents of Mn and ammonia are shown in Table 7. In the geopolymer leaching test, the leaching amounts of Mn were 0.213 mg/L and 0.123 mg/L for day 1 and 28, respectively, and those of ammonia were 0.766 mg/L and 0.538 mg/L for day 1 and 28, respectively. The measured leaching toxicity meets the requirements of the Integrated Wastewater Discharge Standard (GB 8978-1996) [37] (total manganese < 2 mg/L, ammonia nitrogen < 15 mg/L), and the three-dimensional network of geopolymer was capable of encapsulating the contained heavy metals well, demonstrated by the content of heavy metals decreasing with increased curing time. In addition, the heavy metal Mn can replace sodium and calcium in the three-dimensional network structure to realize the curing of free manganese. The alkaline thermal activation removes the ammonia and nitrogen from the geopolymer so that only a small amount of free manganese and ammonia nitrogen are detected in the samples.

### 3.4. Characterization

The content of this section focuses on analyzing the characterization results of EMR, E-GP, and SW-GP. The only raw material for E-GP is EMR, and SW-GP has a coordination ratio of EMR:CG:FA = 0.43:0.34:0.23. Both groups of geopolymers were prepared under the conditions of an exciter modulus of 1.3, L/S = 0.9, and a curing temperature of 60 °C. The results are summarized in the following sections.

#### 3.4.1. Phase Transformation Analysis

**Figure 5:**
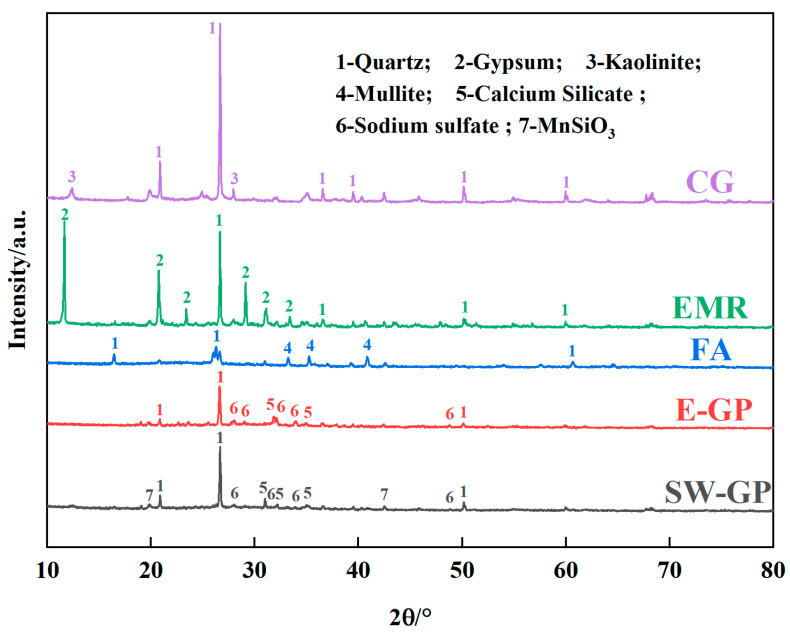
XRD patterns of EMR, CG, FA, E-GP, and SW-GP.

The XRD patterns of EMR, CG, FA, electrolytic manganese slag base polymer (E-GP), and SW-GP are shown in Figure 5. In Figure 5, the EMR is mainly composed of quartz and calcium sulfate dihydrate, the main components of gangue are quartz and kaolinite, and the main substances of fly ash are quartz and mullite. Comparing the XRD patterns of the raw materials, a large amount of diffraction peaks of new crystalline phases appeared in the geopolymer, indicating that chemical bond breaking and material reorganization of the geopolymer to form new phases occurred during the hydration reaction. Among them, the peaks of mullite and calcium sulfate dihydrate were weakened or even disappeared, generating a hydrated calcium silicate gel [38]. In addition, although the diffraction peaks of quartz decreased, the diffraction peaks did not disappear and demonstrated that quartz still existed in the geopolymer, which indicates that part of the quartz had reacted to generate new substances while the other part remained in the geopolymer. Additionally, the quartz that had not been involved in the reaction was able to fill the pores of the silica–oxygen tetrahedral network structure formed in the geopolymer to improve the compressive strength of the geopolymer. Meanwhile, in the geopolymer, sodium sulfate was formed due to the reaction of sulfate with sodium. It has been shown that sodium sulfate accelerates the setting of cementitious materials such as cement and improves early compressive strength to some extent [39]. It is noteworthy that MnSiO_3_ was found in the samples, indicating that Mn^2+^ has entered the framework of the geopolymer and formed new phases, with only a very small amount of free Mn thus detected in the leachate of the geopolymer [40].

#### 3.4.2. Functional Groups Analysis

**Figure 6:**
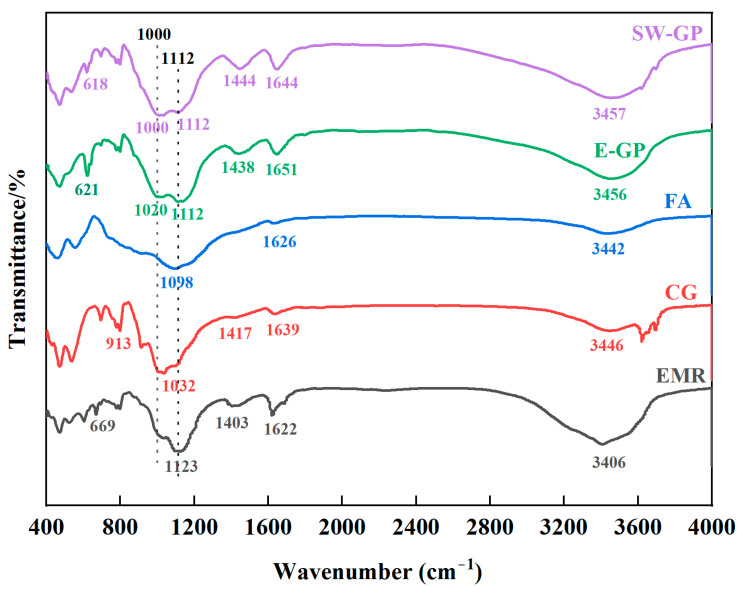
FTIR spectra of EMR, CG, FA, E-GP, and SW-GP.

The changes in chemical groups in raw materials and geopolymers were analyzed and investigated using FTIR, as shown in Figure 6. The peaks at 3457 cm^−1^ and 1642 cm^−1^ are mainly from the asymmetric stretching of hydroxide roots, which indicates the presence of water in the EMR, CG, FA, and geopolymer. From the figure, there is a strong characteristic peak near 1080 cm^−1^ for the EMR, FA, and geopolymer, and this position is usually the characteristic peak of Si-O-Si in quartz material. The absorption peak at 1444 cm^−1^ is mainly from the O-C-O stretching vibration, which is due to the reaction of carbon dioxide in the air with the bases in the geopolymer and the spikes formed due to the asymmetric stretching of CO_3_^2−^. The peak at 1000 m^−1^ is mainly caused by the tensile vibration of the silicate derivative Si-O-T (T = Si or Al), and the peak at 1000 cm^−1^ of the raw material, which corresponds to the peak in the geopolymer, shifted to 1112 cm^−1^, which indicates that a geopolymerization reaction took place and the corresponding gelling substance was produced. The small peaks at 1020 cm^−1^ and 1000 cm^−1^ observed in the E-GP/SW-GP samples can be considered as indicators of CSH, which is expected to be caused by alkali activation [41].

#### 3.4.3. XPS Analysis

**Figure 7:**
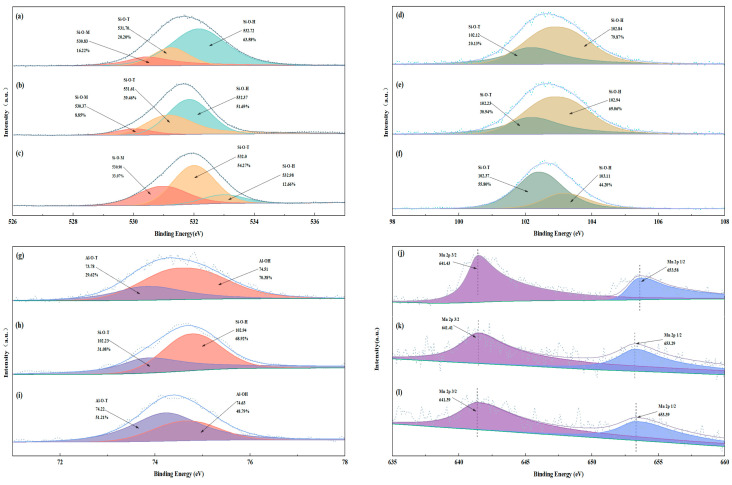
(**a**–**c**) The XPS spectra of O1s in EMR, E-GP, and SW-GP. (**d**–**f**) XPS spectra of Si_2p_ in EMR, E-GP, and SW-GP. (**g**–**i**) XPS spectra of Al_2p_ in EMR, E-GP, and SW-GP. (**j**–**l**) XPS spectra of Mn in EMR, E-GP, and SW-GP.

The XPS results of elemental O in the geopolymers after EMR, E-GP, and SW-GP curing for 28d are shown in Figure 7a–c. The O1s are separated into three peaks and characterized by the chemical bonds Si-O-H, Si-O-T (T = Al or Si), and Si-O-M (M = Na or Mn). In addition, comparing EMR and E-GP, the percentage of Si-O-H in the DGP group decreases significantly, while the percentages of Si-O-T and Si-O-M increase significantly. The decrease in the percentage of Si-OH is due to the geopolymerization process and its continuous conversion to Si-O-T to generate the skeleton structure, which is why the percentage of Si-O-T increases. The increase in the percentage of Si-O-T is due to the Mn and Na entering into the skeletal structure and coordinating with Si-O-T to generate Si-O-Na and Si-O-Mn, and this result is in agreement with the results of XRD analysis. The XPS results of Si_2p_ are shown in Figure 7d–f. From Figure 7b, Si_2p_ is divided into two peaks for Si-O-T and Si- OH. The percentage of Si-O-T increases with curing time, which is mainly due to the increase in the degree of geopolymerization and the conversion of Si-OH to Si-O-T, i.e., geopolymerization from the original silicate structure gradually transforms it to the Si-O-T structure, and the result is in agreement with the FTIR analysis. The XPS results of Al_2p_ are shown in Figure 7g–i. From Figure 7g–i, it can be seen that Al_2p_ is also divided into two peaks, one for Al-O-T and one for Al-OH. Among them, the percentages for Al-O-T and Al-OH content in the E-GP group changed less, indicating that the geopolymerization reaction was not strong enough, whereas the percentages for Al-O-T content in the SW-GP group increased and the percentages of Al-OH content decreased, indicating that a violent geopolymerization reaction occurred, a result consistent with FTIR analysis. The XPS results of Mn_2p_ are shown in Figure 7j–l. From Figure 7j–l, it can be seen that the binding energy of Mn_2p_ 2/3 is around 641.4 eV before and after the reaction. Combined with the change in the binding energy of O1s and the fact that only a very small amount of free Mn was detected in the leaching test, it is hypothesized that during the geopolymerization process, Mn2+ is either wrapped up by the three-dimensional mesh structure of the geopolymer or reacts to form more stable compounds that immobilize the interior of the geopolymer and reduce the leaching concentration of Mn [42].

#### 3.4.4. Micromorphology Analysis

**Figure 8:**
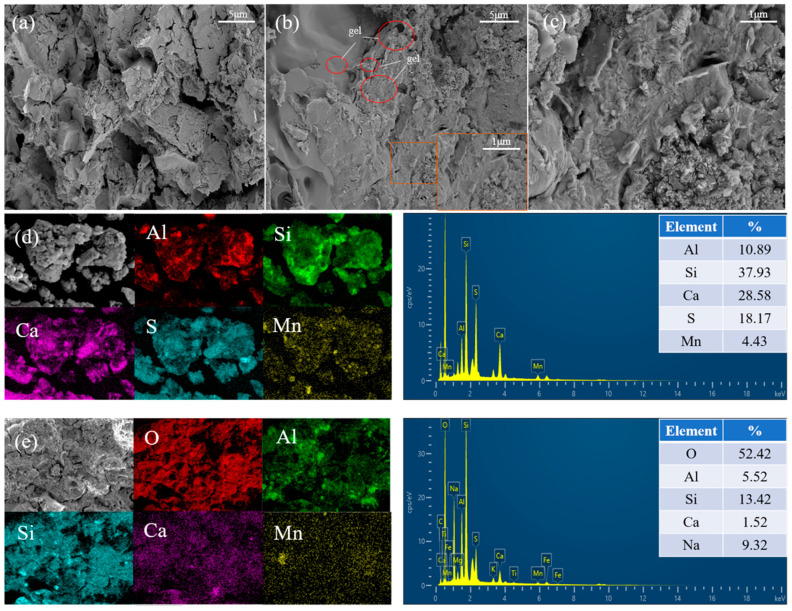
(**a**) SEM image of E-GP; (**b**) SEM image of SW-GP; (**c**) enlarged SEM image of SW-GP; (**d**) EDS results of EMR; (**e**) energy spectrum of SW-GP.

In Figure 8a, the geopolymer prepared only with EMR has a loose internal structure with more pores and poor gelling properties. Figure 8b,c show SEM images of the SW-GP and its magnification, from which the formation of an amorphous gel with fewer pores and a denser structure can be seen, which helps to obtain better compressive strength.

The elemental distribution of Ca, Si, Al, Mn, and S in EMR is shown in Figure 8d. The distribution of Ca and S proves the presence of calcium sulfate, which is consistent with the XRD results. The elemental distribution of SW-GP is shown in Figure 8e, with O, Si, Al, Ca, and Na shown as the major elements in the geopolymer. The elements Si, Al, and Ca combine to form the basic skeleton of the geopolymer. In addition, the same elements of Si and O can be seen in the Mn-enriched areas, and it is hypothesized that Mn reacted with Si and O to form new substances. At the same time, the XRD results show that the Mn in SW-GP reacted to form stable MnSiO_3_. Based on the elemental distributions in the EDS results, it is verified that Mn is encapsulated by the geopolymer network and exists in the geopolymer as a complex of Mn with Si, Al, and Ca.

### 3.5. Reaction Mechanisms

**Figure 9:**
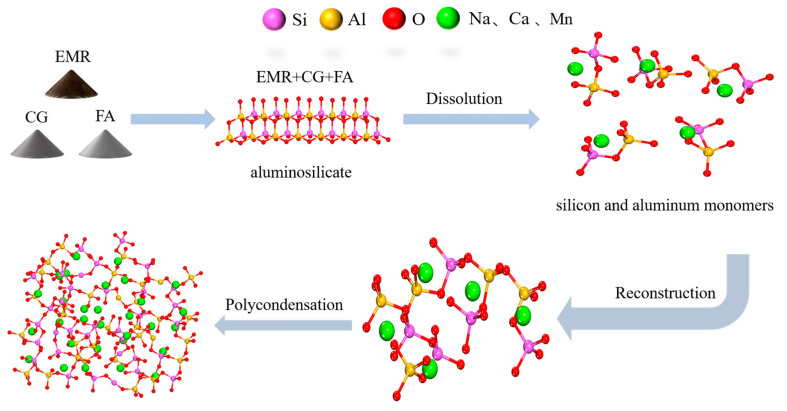
Reaction mechanism of geopolymer formation.

The reaction mechanism is shown schematically in Figure 9. The main minerals of EMR and FA are quartz and mullite, and the main minerals of CG are quartz and kaolinite. Under the action of an alkali alkaline exciter, the solid ASM in the raw material is dissolved, releasing aluminate and silicate. The dissolved silicates and aluminates then form polymer monomers through diffusion, localization, and condensation, and the monomers are then linked by condensation to form a polymer gel [43]. In geopolymers, certain cations such as Na^+^ and Ca^2+^ are present in the voids of the three-dimensional mesh structure to neutralize the charge imbalance due to the partial replacement of Si^4+^ by Al^3+^. At the same time, Mn^2+^ will also be wrapped by the reticular structure of the geopolymer to achieve the effect of immobilization [44]. In addition, the free Mn^2+^ will also generate stable Mn(OH)_2_ under alkaline conditions during the reaction process, and the relevant reaction equation is shown below.

SiO_2_ + 2NaOH→[SiO_3_]^2−^ + 2Na^+^ + H_2_O

Al_2_O_3_ + 2NaOH + 3H_2_O→2Al(OH)_4_^−^+ 2Na^+^

Al_2_O_3_ + 2NaOH→2AlO_2_^−^ + 2Na^+^ + H_2_O

a[SiO_3_]^2−^ + b[AlO_4_]^−^ + nCa^2+^ + zOH^−^ → Ca_n_[(SiO_2_)_a_(AlO_2_)_b_]

a[SiO_3_]^2−^ + b[AlO_4_]^−^ + nMn ^2+^+ zOH^−^ →Mn_n_[(SiO_2_)_a_(AlO_2_)_b_]

Mn^+^+2OH^−^→Mn(OH)_2_

## 4. Conclusions

SW-GP was prepared through excitation with a Na_2_SiO_3_ solution using EMR, CG, and FA as raw materials. Under the conditions of 43% EMR, 34% CG, 23% FA, an exciter modulus of 1.3, a 0.9 L/S ratio, a 60 °C curing temperature, and a 24 h curing time, the strength of the prepared geopolymer reached 12.0 MPa, exceeding the strength of MU7.5 no-burn bricks. The results of toxicity leaching experiments showed that the leaching concentrations of the main heavy metal (Mn) and ammonia nitrogen in geopolymer were 0.123 mg/L and 0.538 mg/L, respectively, which were lower than the national standards. It was shown that EMR, CG, and FA were able to be used as raw materials in the preparation of geopolymer blocks in which a large amount of gelling material was formed (such as geopolymer gels and hydrated calcium silicate gels), and free Mn^2+^ was wrapped by the geopolymer network in addition to the generation of stabilized MnSiO_3_ such that the geopolymer had good mechanical properties and chemical stability. This method can treat and utilize a large amount of EMR, which has high economic and environmental significance.

## Figures and Tables

**Table 1:** Chemical composition of raw materials (wt%).

Raw Materials	SiO_2_	Al_2_O_3_	CaO	Fe_2_O_3_	SO_3_	MnO	MgO	TiO_2_	Na_2_O	K_2_O	LOI
EMR	31.245	9.842	11.804	4.591	24.511	3.859	1.625	0.479	0.841	2.126	8.217
CG	58.744	24.683	0.422	2.732	0.368	0.138	0.552	0.983	0.576	3.576	6.825
FA	47.318	36.675	4.47	5.549	1.024	0.046	0.482	1.351	0.516	0.864	0.857

**Table 2:** Mixing factors and level design.

Factors	The Minimum	The Maximum
A: EMR (wt%)	0.15	0.55
B: Coal gangue (wt%)	0.15	0.55
C: Fly ash (wt%)	0.15	0.55

**Table 3:** Experimental protocol and response values.

Test Number	Proportion (%)			Compressive Strength (MPa)
	A	B	C	Y
1	15	43.05	41.95	17.1 ± 0.35
2	30	15	55	15.1 ± 0.17
3	42.457	15	42.543	10.1 ± 0.12
4	41.475	41.875	16.65	5.6 ± 0.15
5	55	15	30	5.6 ± 0.18
6	27.68	44.998	27.321	11.3 ± 0.29
7	55	30	15	3.9 ± 0.13
8	15	55	30	16.3 ± 0.32
9	55	15	30	5.7 ± 0.28
10	15	55	30	15.9 ± 0.25
11	30	55	15	9.3 ± 0.14
12	35.516	32.143	32.34	8.3 ± 0.20
13	15.945	29.055	55	22 ± 0.22
14	15.945	29.055	55	21 ± 0.24
15	30	55	15	9.5 ± 0.31
16	55	30	15	4 ± 0.16

**Table 4:** The prediction model of mixture design.

Index	Model	Prediction Equation	*p* Value	R^2^
Y	Special Quartic	Y = 2.93 × A + 14.78 × B + 24.85 × C − 10.35 × A × B − 16.59 × A × C − 7.96 × B × C − 496.28 × A^2^ × B × C + 41.73 × A × B^2^ × C − 443.55 × A × B × C^2^	<0.0001	0.9922

**Table 5:** Analysis of variance for compressive strength modeling.

Terms	Quadratic Sum	Freedom	Mean Square	F Value	*p* Value
Model	523.02	8	65.38	244.57	<0.0001
AB	0.33	1	0.33	1.24	0.3022
AC	0.8	1	0.8	2.99	0.1275
BC	0.21	1	0.21	0.78	0.4062
A^2^BC	1.51	1	1.51	5.64	0.4092
AB^2^C	0.082	1	0.082	0.31	0.5962
ABC^2^	1.12	1	1.12	4.19	0.0799
Residual	1.87	7	1.87	/	/
Lack of fit	1.26	2	0.63	5.17	0.0396
Pure error	0.61	5	0.12	/	/
Sum	524.89	15	/	/	/
/	/	/	C.V.% = 4.58	R^2^ = 0.9922	R^2^_adj_ = 0.9883

**Table 6:** The predicted values and optimized proportion of the geopolymer.

Items		Parameters			Predicted Value
		Optimized Direction	Minimum	Maximum	Optimum Proportion
Level	A	in the set value	0.15	0.55	0.43
	B	in the set value	0.15	0.55	0.34
	C	in the set value	0.15	0.55	0.23
Response value	Y (MPa)	Target → 7.5	5	22	7.1

**Table 7:** Mn and ammonia nitrogen leaching toxicity (mg/L) in raw materials and samples.

Items	/	Mn	Ammonia Nitrogen
EMR	/	989	192
SW-GP	1d	0.213	0.766
	28d	0.123	0.538
GB 8978-1996	/	<2.0	<15.0

## Data Availability

Data are contained within the article.
